# Climate change impacts on child and adolescent health and well-being: A narrative review

**DOI:** 10.7189/jogh.14.04061

**Published:** 2024-05-24

**Authors:** Kerrie Proulx, Bernadette Daelmans, Valentina Baltag, Prerna Banati

**Affiliations:** 1 Independent consultant; 2 World Health Organization, Child Health and Development Unit, Department of Maternal, Newborn, Child and Adolescent Health and Ageing, Geneva, Switzerland; 3 World Health Organization, Adolescent and Young Adult Health Unit, Department of Maternal, Newborn, Child and Adolescent Health and Ageing, Geneva, Switzerland

## Abstract

**Background:**

Worldwide, the climate is changing and affecting the health and well-being of children in many ways. In this review, we provided an overview of how climate change-related events may affect child and adolescent health and well-being, including children’s mental and physical health, nutrition, safety and security, learning opportunities, and family caregiving and connectedness.

**Methods:**

In this narrative review, we highlighted and discussed peer-reviewed evidence from 2012–23, primarily from meta-analyses and systematic reviews. The search strategy used a large and varied number of search terms across three academic databases to identify relevant literature.

**Results:**

There was consistent evidence across systematic reviews of impact on four themes. Climate-related events are associated with a) increases in posttraumatic stress and other mental health disorders in children and adolescents, b) increases in asthma, respiratory illnesses, diarrheal diseases and vector-borne diseases, c) increases in malnutrition and reduced growth and d) disruptions to responsive caregiving and family functioning, which can be linked to poor caregiver mental health, stress and loss of resources. Evidence of violence against children in climate-related disaster contexts is inconclusive. There is a lack of systematic review evidence on the associations between climate change and children’s learning outcomes.

**Conclusions:**

Systematic review evidence consistently points to negative associations between climate change and children’s physical and mental health, well-being, and family functioning. Yet, much remains unknown about the causal pathways linking climate-change-related events and mental and physical health, responsive relationships and connectedness, nutrition, and learning in children and adolescents. This evidence is urgently needed so that adverse health and other impacts from climate change can be prevented or minimised through well-timed and appropriate action.

Climate change is a change attributed directly or indirectly to human activity that alters the composition of the global atmosphere, in addition to natural climate variability observed over comparable periods. Climate change is mainly caused by greenhouse gas emissions from natural systems and human activities. This can result in more frequent and intensified extreme climate-related natural disasters, such as storms, floods, extreme heat and wildfires, and more gradual climatic and environmental changes, such as rising temperatures and drought. Climate change also relates to long-lasting changes to landscapes and physical environments caused by rising sea levels and altered ecosystems. Human-induced activities have increased carbon emissions over the last century, accelerating climate change and raising the average global temperature to an alarming level. In 2022, the world encountered 387 climate-related disasters, including storms, floods, wildfires and droughts, which resulted in the loss of 30 704 lives and affected 185 million individuals [1].

The world’s changing climate affects the fundamental rights of children to survive, thrive and reach their full potential. Exposure to climate change-related events during childhood can have long-lasting effects throughout the lifetime. For example, a national USA study found an increased risk of poor mental health and anxiety among adults who experienced an extreme climate-related event before the age of five, such as a tornado, flood, earthquake or hurricane [2]. Furthermore, cohort evidence from Argentina found that exposure to severe climate-related events during childhood reduced the average number of years of schooling and increased the risk of poverty and unemployment in adulthood [3]. Such findings, among others, indicate that research is needed to understand better the interrelationships between climate change and various child and adolescent health and well-being outcomes to help proactively mitigate against potentially negative impacts.

According to the United Nations International Children’s Emergency Fund (UNICEF), half of the world’s children are at ‘extremely high risk’ of climate change impacts due to exposure to multiple climate hazards and a lack of access to essential health and other services that can help children, families and communities to mitigate and recover from climate-related events [4]. In 2022, the year with the highest-ever recorded number of internally displaced people due to climate-related hazards, more than one-third (25.2 million) were children under the age of 18 [5]. Children and adolescents are at particular risk of climate change events because of their rapidly developing brains and bodies, vulnerability to disease, need for nurturing care and limited capacity to avoid threats and impacts. Children and adolescents may also be more likely to fear and worry about climate change than other age groups. Heat stress contributes to significant adverse health outcomes, particularly for infants, young children, and pregnant women. UNICEF has recently developed a technical note for planning and preparedness to protect populations from heat stress [6].

In this narrative review, we outline the available systematic review evidence on the effects of climate change-related events on children’s and adolescents’ health and well-being. Recent reviews have examined climate change’s impact on children’s physical health. The rationale for this review is to take a broader approach to child well-being and not focus solely on physical health effects. Aligning with the World Health Organization (WHO) and UNICEF comprehensive agenda for the health and well-being of children and adolescents [7], this review explores climate change effects across the six domains of health and well-being outlined in this comprehensive agenda, including physical and mental health, responsive relations and connectedness, nutrition, safety and security, and learning opportunities (**Table 1**). The narrative review focuses mainly on extreme weather-related disasters and rising temperatures, as this is where research has been most abundant. Other climate-related events, such as air pollution and drought, are discussed where evidence is available. Alongside these, climate change can have consequences due to loss of land, flight and migration, and conflict, but these are beyond the scope of the current review.

**Table 1:** Domains of child and adolescent health and well-being

Domain	Definition
Good health	This domain highlights the importance of good physical and mental or emotional well-being. To ensure good health, daily needs must be met by health promotion, stress reduction, disease prevention, universal access to preventive, curative, and rehabilitative services, and support for emotional well-being.
Responsive relationships and connectedness	Responsive relationships with caregivers, parents and family members, peers and the community enhance children’s and adolescents’ psychosocial stability and resilience. Responsive caregiving for infants and young children requires engagement, mutually enjoyable interactions, emotional bonding and language.
Adequate nutrition	Adequate nutrition differs by age and stage of life; a newborn, for instance, requires early initiation of and exclusive breastfeeding for the first six months to provide enough nutrition and proper development. After six months of age, they require continued breastfeeding and appropriate complementary feeding for up to at least two years, while older children and adolescents require food choices for an adequate diet that is varied and balanced in nutrients to support growth and development. In addition, food safety and security are critical for adequate nutrition.
Security, safety and a supportive, clean environment	Safety comprises protection from harm or injury, whether intentional or unintentional, including child abuse, violence, and harmful cultural practices and from lifelong emotional, mental and social maladjustment. Safety also consists of access to adequate, stable housing and a clean, safe environment free of risks from air pollution and toxins.
Opportunities for learning and education	Social interactions and play enrich learning and stimulate children’s brain connections from birth. Cognitive, emotional, and social development are stimulated first through strong interactions with the immediate family and the environment. Older children and adolescents should have formal education and training in life skills.
Realisation of personal autonomy and resilience	Personal autonomy is the ability to develop incrementally the capacity to make meaningful choices, have self-esteem and express and direct oneself according to one’s evolving capacities and stage of development. It includes having a sense of purpose, a desire to succeed and optimism about the future. Resilience involves equipping children and adolescents with the ability to handle adversity now and later, persevere, and learn how to cope with adversities.

## METHODS

Following a review of technical documents from the WHO, we identified five critical domains for climate change effects: a) child and adolescent mental health and physical health, b) responsive relationships and connectedness, c) nutrition, d) safety and security, and e) learning opportunities. We did not explore the ‘personal autonomy and resilience’ theme due to insufficient research. However, some of the issues under this theme, such as optimism and coping with adversities, are covered under the mental health theme of eco-anxiety in this paper.

The search strategy commenced with a broad reading of the existing literature and consultation with experts to identify the key areas of interest on how climate change can affect child and adolescent health in these topic areas. We included climate change and health-related terms to identify relevant literature, including terms such as temperature, heat, heatwave, hot, flooding, floods, hurricane, drought, forest fire, thunderstorm, cyclone, tornado, bushfire, pollution, air quality, snowstorm, natural disaster, mental health, mental illness, well-being, anxiety, depression, posttraumatic stress disorder (PTSD), family functioning, parents, caregivers, eco-anxiety, eco-anxiety, asthma, respiratory disease, respiratory illness, diarrheal disease, vector-borne disease, malaria, dengue, malnutrition, stunting, under-weight, violence, abuse, neglect, cognitive development, academic achievement, schooling and learning.

Rather than being exhaustive, we chose this selection to highlight the broad array of climate change factors that likely act to impact child and adolescent well-being. It draws on peer-reviewed evidence primarily from meta-analyses and systematic reviews from 2012–July 2023. We included single studies to describe and illustrate key findings. We searched the Web of Science, PubMed, and Google Scholar. Further, we screened abstracts and full papers for eligibility relevant to how climate change is interconnected with child and adolescent health and well-being. Extra papers of interest were brought to our attention during the writing process by examining paper reference lists and suggestions from collaborators. We included relevant papers published in English.

## RESULTS

### Climate change impact 1: Child and adolescent health

This section focuses on climate change’s effects on children’s mental and physical health. Evidence on the associations between climate change and mental health in children and adolescents cover two key themes: the direct effects of climate events and particularly extreme weather events on children and adolescent’s mental health, including PTSD and other mental health issues, and eco-anxiety and negative emotions among children and adolescents linked to awareness of climate change.

Following the review of mental health effects, this section explores climate change effects on two areas of child and adolescent physical health, where there have been relatively extensive and consistent research findings on the effects of climate-driven changes in air quality on asthma cases and respiratory illnesses in children and adolescents, and increased health risks from food- and water-borne pathogens and vector-borne diseases due to changing temperatures, precipitation patterns, flooding, and other climate events.

#### PTSD and other mental health issues

More frequent, unpredictable, and intense extreme weather events – such as storms, floods and fires – can destroy homes and livelihoods and exhaust overstretched health and other community services. According to multiple systematic reviews [8,9], including a review of studies specifically from low- and middle-income countries [10], prevalence rates for mental health disorders in children and adolescents vary widely in the aftermath of such disasters, ranging from 2–83% for PTSD and 2–66% for depression. The wide variation may relate to the severity of the exposure and children’s experience of loss or threats. In a meta-analysis, Tang and colleagues [8] found that children who experienced injury, fear, or bereavement from a disaster, witnessed injury or death or had poor social support were at greater risk of developing a mental health disorder. Similarly, a review of eight studies of children aged three to 18 years found that disaster exposure and a lack of social support were significant risk factors for developing PTSD following disasters [11]. A review by Ma and colleagues [12] highlighted the importance of social support and family factors (e.g. parenting style) in protecting children and adolescents from developing PTSD, depression and anxiety post-disaster.

A review by Burke and colleagues [13] found elevated rates of internalising behaviours, such as phobias, sleep disruption and attachment disorders following climate-related disasters. These results were further underlined by a meta-analysis by Rubens, Felix, and Hambrick [14], who summed up 62 studies and found increased rates of depression, panic, and anxiety in children and adolescents after disaster exposure and externalising, aggressive behaviour. The authors also found a stronger association between disaster exposure and poor mental health in medium Human Development Index countries compared to high Human Development Index countries. This suggests that children in countries with less financial and other resources may be at greater risk for poor mental health after climate-related disasters.

In summary, reviews provide reasonable evidence for an association between climate-related disasters and disruptions to children’s mental health, with PTSD being the most studied. However, studies have mostly been cross-sectional, making it difficult to draw credible conclusions about attributing mental health problems to disasters, so future research is needed to verify these associations.

#### Eco-anxiety and negative emotions

An emerging body of evidence suggests that indirect exposure to climate change events through media content or from thinking about climate change can evoke strong negative emotions in children and adolescents or eco-anxiety. Reviews, which are based primarily on studies from high-income countries, have found that children and adolescents can experience eco-anxiety and negative affective responses in reaction to awareness of climate change, including depression, anxiety, and extreme emotions like sadness, anger, and fear [15–17]. Females may be more vulnerable to eco-anxiety [15]. Chalupka and colleagues [18] found that children and adolescents in indigenous and subsistence communities may experience grief concerning the actual or anticipated loss of species, ecosystems and meaningful landscapes linked to culture, heritage and livelihoods. Despite a recent increase in interest in eco-anxiety and other negative emotions, there remains a relative dearth of robust empirical data on the prevalence, nature and severity of the mental health impacts related to children and youths’ awareness about the climate crisis. There is a need for efforts to advance conceptual clarity on eco-anxiety and broaden the research base to more diverse populations, including children in low and middle-income countries.

#### Mortality and adverse birth outcomes

The evidence on the mortality impacts of climate change focuses mainly on high temperatures. An overview of systematic reviews [19] and four reviews [20–23] have synthesised the existing literature and found inconsistent results on the association between higher temperatures and childhood (all ages) mortality. However, reviews report a strong association between heat stress and mortality in young children [20,22,23]. For example, a systematic review of 33 studies on the impact of ambient temperature on children’s health found that children under one year of age were at high risk of heat-related mortality [22]. More recently, an individual study drawing on data from the Demographic and Health Surveys Program in 53 countries in Africa, Asia, and Latin America found that very hot (and humid) days generated significant infant mortality effects, particularly during the first month after birth [24]. Notably, the most significant impacts of heat on infant mortality occurred in the more humid regions of Asia rather than in hotter but drier parts of sub-Saharan Africa, suggesting an amplifying threat of heat and humidity to infant survival. In the USA, a study found that each additional 1°C in daily temperature increased the risk of infant mortality by 22.4% [25]. A study from South Africa found that children under the age of five exhibited high mortality risks from heat compared to other age groups [26].

Evidence on climate change and adverse birth outcomes focuses primarily on high temperatures. Reviews report a significant association between exposure to heat during pregnancy and adverse birth outcomes, primarily preterm birth and stillbirth [27–33]. Evidence for heat sensitivity on birth weight is more limited, but most studies report minor changes in birth weight. In a meta-analysis of 47 studies, Pham and colleagues [28] noted that the odds of preterm birth and stillbirths rose by about 5% per 1°C increase in temperature and even higher during heat waves. Heat sensitivity in pregnancy was highest among women with low socioeconomic status. More recently, in a single global study of Demographic and Health Surveys Program data from 14 low- and middle-income countries, McElroy and colleagues [34] found that high daily temperatures and smaller diurnal temperature ranges (correlated with humidity) during the last week before birth increased the risk of preterm birth and stillbirth, particularly among women with lower levels of education and in rural areas.

#### Asthma and respiratory illnesses

Climate change events, mainly through air pollution and wildfire exposure, are likely to worsen air quality and cause or exacerbate respiratory-related adverse health outcomes among children and adolescents. Existing reviews link exposure to air pollutants and climate-driven changes in outdoor particulate matter and ground-level ozone with various adverse health effects in children, including asthma, reduced lung function and other respiratory diseases [19,35–37]. Reviews also indicate that high temperatures reduce air quality and are associated with increased childhood asthma and paediatric respiratory diseases [19,21,38].

#### Pathogens and vector-borne diseases

An overview of systematic reviews [19] on food-borne and water-borne infectious diseases suggests that higher temperatures are associated with infectious bacterial diseases, and children might have higher risks than other age groups. For example, Chua and colleagues [39] analysed 57 articles. They found an increased risk of some bacterial pathogens (e.g. cholera, schistosomiasis, salmonella, *Escherichia coli* gastroenteritis) per 1°C temperature rise, resulting in increased gastrointestinal manifestations, such as vomiting and diarrhoea. In addition, a review found that flooding was associated with increased diarrhoea rates due to disrupting access to safe water and sanitation [40]. Drought reduces the quantity of water, and drought-affected families may resort to using unsafe water, increasing the risk of bacterial infections in children. Children need to consume more food and water per unit of body weight than adults and are more vulnerable to unsafe water sources.

An overview [19] and scoping review of systematic reviews [41] suggest that higher temperature and increased rainfall are associated with some vector-borne diseases, but with few quantifications of the associations specifically for children. Changes in the geographic range and intensity of malaria and dengue transmission under future climate change conditions may spread into regions with immunologically naïve populations and increase the global population living in at-risk areas. For malaria, studies predict a northward shift of the malaria-epidemic belt, and a northward shift is also predicted for dengue. This will affect populations who are currently naive or at low risk and increase the disease burden, both on the northern and southern hemisphere.

### Climate change impact 2: Responsive relationships and connectedness

There are indirect effects of climate change on child and adolescent well-being linked to disruptions in family relationships and functioning, including compromised caregiver capacity for responsive caregiving and caregiver mental health problems following climate-related events. Climate disasters can cause psychological stress among adult caregivers due to trauma and loss of resources (i.e. financial, food, and housing). These added stressors and hardships could negatively impact a caregiver's mental health and capacity for responsive caregiving, resulting in a general deterioration in family relationships and functioning.

According to reviews [42,43] and individual studies [44–46], distress and poor mental health among parents following disasters are crucial drivers in changes to family functioning, with reported increases in family conflict, hostility, and anxious parenting. These indirect effects can increase the risk of PTSD, depression and anxiety in children and adolescents. Social support structures can also be disrupted, leading to isolation from family and friends, religious organisations, community groups, and others, compromising responsive relationships and connectedness. Distress and poor mental health among parents or caregivers are hypothesised to be the key drivers in the changes to family functioning, which was a strong predictor of child posttraumatic stress symptoms.

A single study with very low-income mothers and their toddlers found that financial strain and neighbourhood violence were associated with higher levels of mothers’ depressed mood following Hurricane Katrina, which was linked to less parenting efficacy [46]. Another study found that parents with higher psychopathology and less social support following Hurricane Katrina reported using more maladaptive coping strategies and endorsed greater corporal punishment, which was associated with children’s elevated risk for PTSD post-hurricane [45]. In Puerto Rico, 18 months after a hurricane, a study of exposed families reported poorer parent-child relationship quality, parent-child involvement, and parental relationship quality than non-exposed families [47]. Following a severe cyclone in Australia, families reported more anxious parenting (i.e. more protective, less granting of autonomy, more communication of danger), especially caregivers experiencing symptoms of depression or anxiety [48]. These findings suggest that responsive caregiving, connectedness and caregiver mental health are central factors in child health and well-being outcomes post-disaster. Extreme weather events can directly impact caregivers' mental health via disaster-related trauma or indirectly affect mental health through financial strain and loss of resources, increasing the risk of poor parenting.

### Climate change impact 3: Child and adolescent growth and nutrition

Reviews provide robust evidence that high temperatures and drought are associated with food insecurity, reduced dietary diversity, wasting, stunting and being underweight, especially for rural children [49,50]. Wasting and being underweight are more prevalent in the weeks and months following an extreme weather event, but stunting is more common years afterwards. A single study drawing on Demographic and Health Surveys Program data from 18 countries in sub-Saharan Africa found that hot-and-dry conditions are associated with reduced child weight [51]. In South Asia, extreme precipitation significantly decreased children’s height-for-age, primarily in households that lacked safe sanitation and had low levels of education [52]. Such climate-related events can disrupt agricultural production, leading to crop failure, death of livestock and income loss, which can lead to food insecurity and rising food prices. Food supply shortages and income loss can lead to undernutrition, especially during the first two years of life, affecting physical and cognitive development. Lastly, there is emerging evidence of a link between poor air quality and reduced child linear growth during early childhood [53].

### Climate change impact 4: Child and adolescent security and safety

Safety and security include protection from physical dangers and emotional stress. This section focuses on the evidence base related to the association between climate events and children’s exposure to violence and abuse. A systematic review and meta-analysis examining the association between climate disasters and violence against children identified 11 quantitative studies, primarily cross-sectional designs [54]. The authors found no consistent association between extreme weather events and violence against children but noted that more high-quality and nuanced research is needed. Similarly, a systematic review of 22 studies on violence against children in humanitarian contexts, including climate disasters, concluded that the current body of evidence offers an incomplete picture regarding the prevalence, nature, and impact of violence against children in climate emergencies [55].

On the other hand, reviews have found that extreme weather events can be associated with increases in gender-based violence, particularly intimate partner domestic violence, towards women and adolescents [56,57]. Violence against women and violence against children tend to co-occur in households. Witnessing domestic or intimate partner violence negatively impacts children’s mental health and well-being. Ultimately, more robust research is needed to understand better the associations between climate change and children’s safety and security, including types of violence that may increase, for which groups of children and adolescents, and in what contexts. Lastly, a review of 24 articles found that climate-related crises worsen known drivers of child marriage and can push families to marry their daughters early through loss of assets and opportunities for income generation, displacement, educational disruption, and the creation of settings in which sexual violence and the fear of sexual violence increase [58].

### Climate change impact 5: Child and adolescent learning opportunities

We could not find a systematic review of the associations between climate change and educational or learning outcomes in children and adolescents. This one has been the most under-researched of all the domains in this paper. According to a single study that included a 58-country assessment, each additional day above 26.7°C (80°F) during the three years preceding an exam lowered scores by 0.18% of a standard deviation, with the effect more significant for lower-income populations [59]. In another study drawing on census data from 29 countries, Randell and Gray [60] found that experiencing hotter-than-normal conditions during childhood was associated with fewer years of schooling and lower educational attainment in some parts of the world. Hot classrooms can be distracting and unmotivating, which can increase student absenteeism. Furthermore, heat can interfere with sleep quality, cognitive function and the ability to concentrate, reducing learning outcomes.

Another set of single studies showed that flooding and rainfall shocks are associated with poorer cognitive ability, lower school enrolment, reduced grade completion, and increased child labour [61–63]. For example, a study from Mexico highlights that the 1997–98 El Niño reduced families’ income and led to stressful conditions for many poor children and families. About five years later, children exposed to El Niño posted test scores in language development, memory and visual-spatial thinking that were 11–21% lower than children of similar age unaffected by the shock [64]. Adverse physical effects were also found, including lower height, higher propensity to stunting and lower weight for age compared to the control group, which may be linked to reduced income and food security due to El Niño. The loss of family resources may disrupt education and negatively impact schooling outcomes.

## DISCUSSION

In this paper, we reviewed the evidence on the associations between climate change-related events and children’s and adolescents’ health and well-being. Much of the evidence is based on cross-sectional and observational designs, showing correlations between climate events and pertinent health outcomes for children and adolescents. The available evidence points to consistent and important findings on the associations between changes in climate and child and adolescent health on four themes.

First, there is robust systematic review evidence illustrating that extreme climate-related disasters are associated with PTSD and other mental health disorders, mainly where there has been exposure to disaster-related injury, death and loss, and there is a lack of social and family support. Children and adolescents also have a heightened risk of internalising and externalising behaviours following climate-related disasters. Eco-anxiety is an emerging topic, with recent reviews indicating that negative emotions in children and adolescents may be associated with awareness of climate change.

Second, regarding physical health, there is clear evidence that climate-driven changes to air quality are associated with asthma and respiratory illnesses. High temperature and flood exposure are associated with greater exposure rates to waterborne pathogens, increasing diarrheal diseases in children. High temperatures and changes in precipitation patterns may expand the ranges and active-season lengths of mosquitoes and other organisms that carry vector-borne diseases, potentially increasing exposure to malaria, dengue and other diseases. Third, evidence shows that high temperatures and drought are associated with reduced dietary intake and children’s malnutrition. Poor air quality is also linked to reduced child growth. Fourth, family stability and resources play an essential role, and children are more likely to display PTSD and other mental health issues where there is poor caregiver mental health and disruptions in responsive caregiving and family functioning post-disaster.

In contrast to the above, evidence of violence against children in climate-related disaster contexts is inconclusive, primarily due to a lack of good-quality studies. Therefore, this is an area where good methodological studies are required, especially given that evidence suggests domestic violence is associated with climate disasters. Lastly, limited systematic review evidence exists on the associations between climate change and children’s learning. Evidence indicates that high temperatures may harm learning and school achievement. Flooding and extreme rainfall also appear to be associated with reduced learning outcomes. Climate-related displacement also affects education. Unlike refugees, climate-displaced children and adolescents have no specific right under international law to residency or education. Barriers include saturated school capacity, destroyed infrastructure, linguistic barriers, exacerbated poverty and dropout, non-recognition of past qualifications, and discrimination [65]. The actual impact of these barriers to education needs more research.

This paper intended to provide a snapshot of how climate change-related events may affect different domains of children’s and adolescent’s health and well-being. It is not a systematic review and should not be interpreted as such. However, we would argue that there is value in bringing together the diverse literature on climate change’s effects on children’s comprehensive health and well-being. The five domains reviewed have typically been studied separately in existing published reviews. The review showed how much remains unknown about the effects of climate change on children and adolescents, direct and indirect, in the short and long term. In particular, limited systematic or empirical work has examined the causal pathways and processes linking climate-change-related events and mental and physical health, responsive relationships and connectedness, nutrition, and learning in children and adolescents. This evidence is urgently needed so that adverse health and other outcomes from climate change can be prevented or minimised through well-timed and appropriate action. Further analyses should incorporate a broader population to understand better how different socioeconomic factors can protect or worsen effects in different child and adolescent health and well-being outcomes than those measured in this report. Lastly, qualitative studies can help better understand how climate-related events impact children and families.

## CONCLUSIONS

Climate change is a most serious threat to the health and well-being of children and adolescents. Its sequelae affect both the physical and mental health of individuals in the first two decades of life as well as family functioning. Evidence about the amplitude and magnitude of harmful effects is still emerging. However, available information demonstrates that unless policies and actions to mitigate climate change are implemented with urgency, gains made in improving child and adolescent health outcomes can dramatically stagnate or be reversed. More research is needed to strengthen the understanding of how climate-related adverse events affect neonatal. child and adolescent health outcomes. In the meantime, adaptation in health services to recognise and address the effects of climate change is a pathway towards sustaining quality of care and raising community awareness and action.
